# Access Control based on Attribute Certificates for Medical Intranet Applications

**DOI:** 10.2196/jmir.3.1.e9

**Published:** 2001-03-17

**Authors:** Ioannis Mavridis, Christos Georgiadis, George Pangalos, Marie Khair

**Affiliations:** ^1^Informatics LaboratoryComputers DivisionFaculty of TechnologyAristotle University of ThessalonikiThessalonikiGreece; ^2^Department of Computer ScienceFaculty of Natural and Applied SciencesNotre Dame UniversityLouaizeLebanon

**Keywords:** Computer security, Medical records systems, computerized, Distributed access control, Attribute certificates, Digital certificates

## Abstract

**Background:**

Clinical information systems frequently use intranet and Internet technologies. However these technologies have emphasized sharing and not security, despite the sensitive and private nature of much health information. Digital certificates (electronic documents which recognize an entity or its attributes) can be used to control access in clinical intranet applications.

**Objectives:**

To outline the need for access control in distributed clinical database systems, to describe the use of digital certificates and security policies, and to propose the architecture for a system using digital certificates, cryptography and security policy to control access to clinical intranet applications.

**Methods:**

We have previously developed a security policy, DIMEDAC (Distributed Medical Database Access Control), which is compatible with emerging public key and privilege management infrastructure. In our implementation approach we propose the use of digital certificates, to be used in conjunction with DIMEDAC.

**Results:**

Our proposed access control system consists of two phases: the ways users gain their security credentials; and how these credentials are used to access medical data. Three types of digital certificates are used: identity certificates for authentication; attribute certificates for authorization; and access-rule certificates for propagation of access control policy. Once a user is identified and authenticated, subsequent access decisions are based on a combination of identity and attribute certificates, with access-rule certificates providing the policy framework.

**Conclusions:**

Access control in clinical intranet applications can be successfully and securely managed through the use of digital certificates and the DIMEDAC security policy.

## Introduction

Today's healthcare environments use clinical electronic records that are shared between computer systems and which may be distributed over many locations and between organizations, in order to provide information to internal users, to payers and to respond to external requests. With increasing mobility of populations, patient data is accumulating in different places, but it needs to be accessible in an organized manner on a national and even global scale. Large amounts of information may be accessed via remote workstations and complex networks supporting one or more organizations, and potentially this may happen within a national information infrastructure [1].

Security is therefore a major concern for managing the electronic healthcare record (EHR).

According to a recent report, more than 1000 accidental deaths have been attributed to computer system failure [2]. No patient's life should be endangered because of information system error or illegal modification of medical information. Equally important is the need to protect patients' privacy by allowing only authorized users to gain access medical records (especially sensitive information). Robust encryption algorithms can be used to protect information as it moves through distributed networks, but the risk of security breaches are now coming from the huge numbers of people needing access, difficulties in evaluating their clearances as well as the challenge of maintaining the integrity and trustworthiness of their sites.

Internet technologies were designed to optimize information sharing not security. Recent efforts to preserve a satisfactory level of Internet security rely on public-key cryptography and digital certificates. A Public-Key Infrastructure (PKI) supports the issuing and management of digital certificates which identify and authenticate authorized users.The emerging complementary Privilege Management Infrastructure (PMI) can provide another type of certificate particularly suitable for authorization purposes. We have already developed the DIMEDAC (DIstributed Medical Database Access Control) security policy, which has been well accepted in healthcare environments. This paper gives an overview of digital certificates for controlling access in distributed medical database systems and proposes a method of access control using digital certificates and DIMEDAC.

## Access management methods

There is a need to change from the specialized case-by-case access management systems in use today and move towards a small number of general approaches that can be operated by individual access management infrastructures [3].

Use of a networked resource is generally controlled by authenticating and authorizing particular users and uses. Authentication is the process where a networked user establishes a right to an identity. A large number of techniques may be used to authenticate a user: passwords, biometric techniques, smart cards, and digital certificates. Authorization is the process of determining whether an identity is permitted to access a resource. An identity has associated attributes, such as permission to access particular resources, or they may be demographically based, and these attributes may change. Authentication and authorization decisions can be made at different points, by different organizations.

A well-known major access management approach to establishing identity is the credential-based approach, with users needing to present some form of evidence that they are indeed the identity claimed. Traditionally the collection of the credential and its validation have been packaged into the application itself, in the familiar 'userid - password' form. However in a network environment, credentials, which are built into protocol mechanisms, such as the use of certificates with HyperText Transfer Protocol (HTTP) and Secure Socket Layer (SSL) Internet protocols are more suitable.

Certificate-based credentials offer a number of advantages. From the user's perspective, this approach facilitates access, minimizes redundant authentication interactions and provides a single sign-on and a user-friendly view of the available resources. From the administrator's viewpoint, it does not require a vast amount of ongoing maintenance. Certificate-based access management provides strong authentication and gives confidence that systems are secure. It is suitable for fine-grained access control and it guarantees user privacy and confidentiality. It also addresses user accountability, with administrators able to investigate if improper use is discovered.

### Access management over the Internet

The benefits of the Internet as a communication medium for sensitive medical information must be tempered with a significantly greater risk to the confidentiality and integrity of information. Security risks cannot be entirely removed when transmitting information over the Internet [4], since the TCP/IP (Transmission Control Protocol/Internet Protocol) allows information to pass through intermediate computers. This makes it possible for a third party to interfere with communications in the following ways:

- *Tampering*: Information in transit is changed or replaced and then sent on to the recipient.
- *Eavesdropping*: Information remains intact, but its privacy is compromised.
- *Impersonation*: Information passes to a person who poses as the intended recipient. The term spoofing is also used to describe the case in which an entity pretends to be someone else.

Fortunately, some well-established techniques and standards collectively known as public-key cryptography (PKC) make it reasonably simple to take precautions. PKC uses a pair of keys, a public key and a private key. Each public key is published, and the corresponding private key is kept secret. Data encrypted with the public key can be decrypted only with the private key and vice versa. PKC and related techniques provide the additional capabilities:

- *Encryption* and *decryption* allows two communicating sides to transform information they send to each other. The sender encrypts, or scrambles, information before sending it. The receiver decrypts, or unscrambles, the information after receiving it. While in transit, the encrypted information is incomprehensible to an intruder.
- *Tamper detection* allows the recipient of information to verify that it has not been altered in transit. Any attempt to modify data or to substitute a false message for a legitimate one will be detected.
- *Source authentication* allows the receiver of information to confirm its origin. That is to verify the sender's identity.
- *Non-repudiation* establishes proof that the sender sent the data and the receiver received it.

By themselves, encryption and decryption do not address the problems of tampering and impersonation. Tamper detection relies on a mathematical function called a one-way hash (also called a message digest). The encrypted hash of the data, along with other information, such as the name of the hashing algorithm, is known as a digital signature. The importance of a digital signature is comparable to the significance of a hand-written signature. Once someone has 'digitally' signed some data, it provides a high degree of non-repudiation. Confirming the identity of the signer, however, also requires some way of confirming that the public key really belongs to a particular person or entity. Digital identification documents called certificates, which are described below, address the issue of impersonation.

## Types of digital certificates

### Identity Certificates

Until recently, the only widely adopted digital certificate has been the identity certificate. An identity certificate (IC) is an electronic document used to recognize an individual, a server, or some other entity, and to connect that identity with a public key. Like a credit card, a passport, or other personal IDs, a certificate provides generally recognized proof of identity. Identity certificates are issued by certificate authorities (CAs) in much the same way as government agencies issue passports after verifiying an individual's identity. Certificate authorities can be either independent third parties or organizations running their own certificate-issuing server software. The certificate authority generally uses published verification procedures to ensure that an entity requesting a certificate is in fact who it claims to be.

When a certificate authority issues an identity certificate, it binds a particular public key to the name of the entity identified in the certificate (such as the name of a doctor). Public-key cryptography uses certificates to help avoid the use of impersonation through forged public keys. Access will only work when the certificate's public key matches with the corresponding private key of the entity identified in the certificate. In addition to a public key, a certificate always includes the name of the entity it identifies, an expiration date, the name of the certificate authority that issued the certificate and other information. Most importantly, a certificate always includes the digital signature of the issuing certificate authority. The certificate authority's digital signature allows the certificate to be used as a 'letter of introduction' for users who trust the certificate authority, but who are not familiar with the entity identified by the certificate.

### Attribute certificates

More recent research and development efforts have resulted in a second kind of digital certificate, the attribute certificate [5,6,7]. An attribute certificate (AC) has a data structure comparable to an identity certificate. However, a major difference is that an attribute certificate does not contain a public key. It contains attributes that specify access control information associated with the AC holder (such as group membership, role, security clearance). Attribute certificates are able to support and implement a significant part of the authorization process. The basic idea is that not all access control decisions are identity-based. Role-based, rule-based and rank-based access control decisions require additional information. For example, information about a user's current role (e.g. physician) or a client's ability to pay for a resource access may be more important than the client's identity.

Although this kind of authorization information can be placed in extension fields of identity certificates, there are two fundamental reasons against doing this. Firstly, the certificate authorities who issue the identity certificates are not usually responsible for this sort of authorization information. As a result, certificate authorities must take additional steps to obtain access control information from the source. Secondly, the authorization information may give different lifetimes for the binding of the identity and the public key. Placing access control information in an IC extension shortens the lifetime of an identity certificate, while time/validity requirements for attribute certificates allow both long-lived and short-lived ACs. Typical validity periods might be measured in hours, as opposed to months and years for ICs [5]. Short validity periods also allow ACs to be used without a revocation mechanism which means access permissions can be changed in a relatively flexible way. Longer-lived ACs may be particularly relevant for authorizations that are relatively static [6]. In this case, if a common authority is responsible for identity and attribute certification, the attributes can be included within IC extension fields, but in most cases it is better to keep the authorization attributes separate from ICs.

In an AC, attributes also need to be protected in a similar way to an IC. Since they are simply digitally signed sets of attributes, ACs provide this protection. Attribute authorities are the entities authorized by one or more users to create and sign attribute certificates [7]. Just like certificate authorities, attribute authorities are responsible for their certificates during their whole lifetime, as well as issuing them.

The attribute certificate format allows any non identity-based authorization information to be bound to an identity certificate by including, in a digitally signed data structure, a reference (linkage) back to a specific identity certificate.

### The proposed access-rule certificates

Traditionally, authorization policies have been expressed and managed in a relatively centralized manner: one person or organization administers and enforces the access control requirements. In many cases however, policy control has to be decentralized. In distributed computing environments, there may be multiple, independent and geographically spread entities (individuals, organizations, institutes, notaries etc.) with authority to control access to their local resources. Each of these parties is responsible for defining access-rules to protect resources and each brings its own set of concerns. In order to address those problems in our approach we propose the use of another kind of digital certificate, the access-rule certificate (RC).

An access-rule certificate has a data structure comparable to an IC and AC. It enables parties responsible for policy to create and distribute access control mechanisms remotely and securely and to create rules authorizing access to their resources. RCs are digitally signed sets of rules. Similar to attribute and certificate authorities, a rule authority (RA) is the entity authorized by one or more users to create and sign access-rule certificates. Rule authorities may be both physically and organizationally remote from the information resource.

A suitable security policy is needed to fully exploit digital certificates in protecting clinical intranet applications. Our DIMEDAC (DIstributed Medical Database Access Control) security policy has the required structure and is PKI and PMI compatible.

## The DIMEDAC security policy

The DIMEDAC security policy provides a role-based authorization mechanism for accessing medical records. Authorization depends on the particular values of the identity-based context parameters of users that form the user location. Furthermore, it provides mandatory security features [8]. The following are some major characteristics of DIMEDAC, which are particularly useful for distributed access control through digital certificates.

### Location control

In distributed medical database systems it is often critical to control the location where users' access requests originate.

A *user location* can be viewed as depending on the following parameters:

- *Site*, which could be a workstation (including the hardware, software and network connection) from where a user logs in the system. It can be of any size or computational power and connected either by cable or wireless.
- *Administrative domain*, which is a part of an organization where a unique administration policy is in effect [9]. Possible types of administrative domains in medical applications might be: clinic, department, hospital, national, international (e.g. European Union).
- *Context parameter*, which differentiates between the need-to-know requirements of users. For example, the identities of particular patients, who have been admitted and charged to particular users with corresponding medical or ward user roles, may be considered as user locations.

Location control is used for two main reasons. Firstly the trustworthiness of the user location (site/workstation or administrative domain) and the user's profile determine the set of roles that a particular user can activate. Secondly, knowledge of the user location (administrative domain or context) also contributes to the decision of the type of access allowed. Control determined by context is highly relevant in the healthcare environment, where there are often temporary increased need-to-know requirements for special cases [10].

As a general rule, the privileges of users are reduced as they are 'located' further from the data.

### The distributed access control mechanisms

The access control mechanisms of the DIMEDAC security policy consist of hyper node hierarchies for user roles, data sets and user locations respectively, as well as sets of user location-dependent authorization rules.

### Hyper node hierarchies

Hyper node hierarchies (HNHs) are used as normal role hierarchies [11] for permission inheritance, as well as to derive security labels. These labels consist of a security level and a set of categories, due to the two types of connections used between nodes [12]. The mechanism of HNH is used to construct the appropriate user role, data set and user location hierarchies.

The user role hierarchy (URH) consists of nodes, which represent the user roles of a specific application, placed at their corresponding classification levels. The number of levels in URH is predefined depending on the granularity of the control needed.

The data set hierarchy (DSH) consists of nodes, which represent the data sets of the application, placed at their corresponding sensitivity levels. The number of levels in DSH is also predefined.

The user location hierarchy (ULH) is a means for representing the organizational structure of the health care establishments involved in the application.

### Location-dependent authorization rules

For access control in wide distributed information systems with multilevel administrative domains, we propose the use of global and local user roles, data sets and user locations. The activation of a global user role determines the ability of the user to access a number of data sets in another remote site. This means that every local security administrator may be able to decide about the authorization of subjects (users) of its administrative domain on objects of other domains. It is clear that access control for other administrators must be limited when they are in other administrative domains [13] and it follows that the privileges of a given user role must be reduced for remote access. This can be accomplished on the basis of user locations, by eliminating the global user role permissions set to access the database remotely. To achieve this a third dimension, concerning the user location, is introduced in the classical access matrix. The resulting access matrix is called Three-Dimension Access Matrix (3DAM) and can be implemented as multiple access matrices, one for each possible user location. A user role (UR) in a user location (UL) has the authority to access a data set (DS) with an access-mode (AM). An authorization rule of this kind can be expressed with a quadruple: {UR, UL, DS, AM}. Data sets in a 3DAM can be defined as data views (e.g. by using the SELECT statement of the Structured Query Language - SQL). Using views of the relational model results in a view-based protection [14]. A significant advantage is a flexible granularity for the definition of objects to be protected. So, it is easy to introduce detailed specifications for specific items (e.g. fields) as well as more general declarations for groups of data sets (e.g. tables) in order to save storage space. The need-to-know requirements of users like doctors are highly dependent on their specific patients. As a consequence, there is a need for increased temporary access privileges for accessing the medical records of their patients. However, there is no way to express this functionality by using static entries in a conventional access matrix. So, we propose, in the context of a view-based protection, the use of dynamic entries that are defined including parameters (e.g. by using ORACLE Dynamic SQL [15]). Such an entry for the table of patients could have the following form:


                    
SELECT patient_fielda, patient_fieldb 
FROM patient_table 
WHERE patient_id IN ':set_of_user_locations'

                

The value of the parameter ':set_of_user_locations' represents the set of locations of the user that requests to access the database. The patient identification code (patient_id) could be used as a global location (e.g. the patient charged to the doctor who is trying to access the database remotely) given that a unique code identification system is in effect.

### Definition of distributed access control mechanisms

The DIMEDAC security policy permits the enforcement of access control policies in large scale distributed systems that are spread over different organizations. It provides a satisfactory level of security of global objects that are accessed by global subjects in a predefined manner.

In the context of a multi-level hierarchy of administrative domains, the following actions should be accomplished for the definition of the access control mechanisms of DIMEDAC (URH, DSH, ULH and 3DAM) in every administrative domain:

- Inheritance of mechanisms from the ancestor (upper level) administrative domain,
- Refinement of those mechanisms in order to meet the specific local needs of the particular administrative domain.

A specific method for the definition of the DIMEDAC access control mechanisms has already been proposed in [8]. This method results in a combination of local and global access control policies, which remain compatible between different organizations without sacrificing the flexibility to further define inner components and to assign more specific authorization rules. The defined access control mechanisms could be stored in special security servers that provide directory and certification services. The process of inheriting the access control mechanisms between different levels of administrative domains could also be accomplished by using access-rule certificates (RCs). As shown in Fig. 2, a security server is proposed to update the inherited URH, DSH, ULH and 3DAM by acquiring specific RCs from the corresponding security server of its upper level, which acts as an access rule authority (RA).

## The proposed operational architecture

The proposed operational architecture consists of two phases. Initially, every user obtains an identity certificate. When users initiate a new session, they must first identify and authenticate themselves by using their identity certificates. Then they activate a subset of user roles and locations, which form a session-dependent user profile that is recorded in a set of short-lived attribute certificates. In subsequent user access requests, an access decision-making process takes place based on the combination of identity and attribute certificates of the user. The proposed operational architecture is described in more detail below.

### Phase 1: Gain Security Credentials

**Figure 1:**
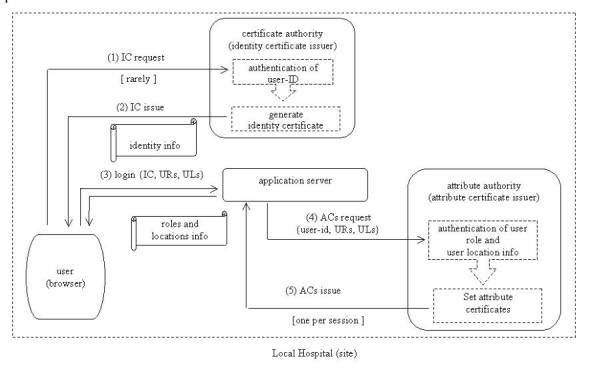
Security credentials acquisition (Phase 1)

As shown in Figure 1, the first phase consists of five steps:

Step 1: Users place their identity certificate request to the local certificate authority (CA) along with their personal data needed for authentication purposes.

Step 2: The local CA authenticates users against their presented personal data, generates the identity certificate and issues it to the user. Conditions for steps 1 and 2 are rarely met.

Step 3: When users initiate a new session, they must first identify and authenticate themselves, during a SSL session with the local application server, by using their IC and password. The validated IC of the user is then used for subsequent remote identification and authentication processes. Then the user activates a subset of user roles (URs), from a set of initially assigned roles, which are needed to accomplish specific tasks. The site of the user is assigned as the initial set of user locations (ULs). The set of user locations is enriched depending on the responsibility/authority of the roles activated by the user. During the upgrade process of the user location set, all the relative administrative domains are included, as well as the context parameters (e.g. the IDs of the patients of the user) of the user task. The user identity, as well as the sets of activated user roles and assigned user locations, forms a session-dependent user profile.

Step 4: The result of the previous step is the preparation of an attribute certificate (AC) request, which is then submitted to the local attribute authority (AA).

Step 5: The AA authenticates the information included in the user profile, sets the attribute certificate and issues it to the user. The issued AC is then used in subsequent access requests during the same session. An attribute certificate is session-dependent and is valid only during the current session. This can be achieved by issuing certificates with a lifetime only a few hours.

### Phase 2: Access Medical Data

**Figure 2:**
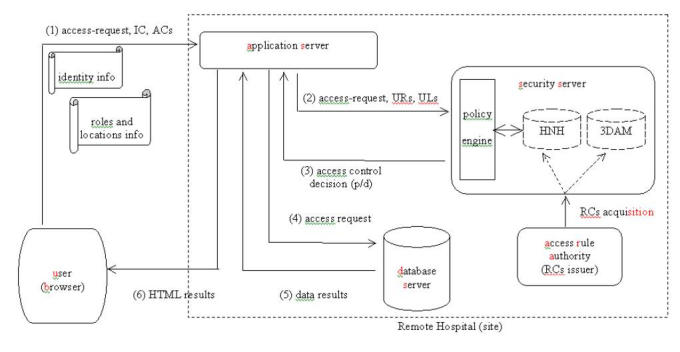
Access medical data (Phase 2)

Subsequently users request access to medical data at any particular site in the whole Intranet. The proposed steps are performed in the following order (Fig. 2).

Step 1: Using the browser, users send their access requests and their accompanying identity and attribute certificates to the application server of a different (remote) site.

Step 2: Using the identity certificate, the remote application server identifies and authenticates the user. Then, it extracts the role (URs) and location (ULs) information from the set of attribute certificates of the user. After a filtering process a new user profile to be used in the remote site is specified containing the final sets of activated roles (URs) and assigned locations (ULs). This user profile is stored in the remote application server and is updated in subsequent user access requests, with new insertions in the presence of new attribute certificates and deletions when the activated user roles and the assigned locations are expired. The new profile and the access request of the user are forwarded (pushed) to the security server of the remote site.

Step 3: The remote security server contains the access control mechanisms as well as the policy engine for the implementation of the DIMEDAC security policy at the remote site. Based on them, a decision making process is performed. The final decision on accessing global data sets at the remote site has the form of a simple 'permitted' or 'denied'.

Step 4: The access decision is returned to the remote application server. Then, the permitted access request of the user is forwarded to the remote database server.

Step 5: The database engine performs the user access request and the data results are returned to the application server.

Step 6: The application server forms the data results in HTML format and sends them back to the user in the form of Web pages.

### Conclusions

This paper contributes to the discussion of an important, and as yet not fully solved, challenge for healthcare: how to exploit Internet technologies to improve quality of care, while protecting patient privacy and the confidentiality and integrity of sensitive medical data. The focus here is on authentication mechanisms using digital certificates not only for user identification, but also for access control decisions and authorizations.

Access control in clinical intranet applications can be successfully achieved by the proposed use of the PKI and PMI infrastructures together with our DIMEDAC security policy. In our implementation approach we use three types of digital certificates: identity certificates (identity-based, long-lived digital certificates with revocation mechanisms) for authentication as well as attribute (role and location-based, short-lived certificates without revocation mechanisms) and access-rule (access rule-based, long-lived certificates with revocation mechanisms) certificates for authorization purposes. The use of identity certificates offers strong authentication, giving all parties confidence in the security and functionality of the system. It is also suitable for fine-grained access control, it guarantees user privacy and confidentiality and it is capable of ensuring user accountability. Attribute certificates also provide a means for exchanging user profiles between different healthcare institutions in a secure way. The access control policy can be securely and effectively propagated with the proposed access rule certificates.

## Abbreviations

3DAM: three-dimension access matrix
A: attribute authority
AC: attribute certificate
AM: access-mode
CA: certificate authority
DIMEDAC: Distributed Medical Database Access Control
DS: data set
DSH: data set hierarchy
EHR: electronic healthcare record
HNH: hyper node hierarchy
HTTP: HyperText Transfer Protocol
IC: identity certificate
ID: identification/identity
PKC: public-key cryptography
PKI: public-key infrastructure
PMI: privilege management infrastructure
RA: rule authority
RC: access-rule certificate
SQL: Structured Query Language
SSL: Secure Socket Layer
TCP/IP: Transmission Control Protocol/InternetProtocol
UL: user location
ULH: user location hierarchy
UR: user role
URH: user role hierarchy
